# Malaria parasite clearance rate regression: an R software package for a Bayesian hierarchical regression model

**DOI:** 10.1186/s12936-018-2631-8

**Published:** 2019-01-05

**Authors:** Saeed Sharifi-Malvajerdi, Feiyu Zhu, Colin B. Fogarty, Michael P. Fay, Rick M. Fairhurst, Jennifer A. Flegg, Kasia Stepniewska, Dylan S. Small

**Affiliations:** 1 Department of Statistics, The Wharton School, University of Pennsylvania, Philadelphia, PA USA; 2 The Graduate Group in Applied Mathematics and Computational Science, University of Pennsylvania, Philadelphia, PA USA; 3 MIT Sloan School of Management, Massachusetts Institute of Technology, Boston, MA USA; 4 National Institute of Allergy and Infectious Diseases, Maryland, MD USA; 5 School of Mathematics and Statistics, The University of Melbourne, Melbourne, Australia; 6 Worldwide Antimalarial Resistance Network (WWARN) and Centre for Tropical Medicine, Oxord, UK

**Keywords:** Bayesian methods, Hierarchical linear models, Clearance rate, *Plasmodium falciparum*

## Abstract

**Background:**

Emerging resistance to anti-malarial drugs has led malaria researchers to investigate what covariates (parasite and host factors) are associated with resistance. In this regard, investigation of how covariates impact malaria parasites clearance is often performed using a two-stage approach in which the WWARN Parasite Clearance Estimator or PCE is used to estimate parasite clearance rates and then the estimated parasite clearance is regressed on the covariates. However, the recently developed Bayesian Clearance Estimator instead leads to more accurate results for hierarchial regression modelling which motivated the authors to implement the method as an R package, called “bhrcr”.

**Methods:**

Given malaria parasite clearance profiles of a set of patients, the “bhrcr” package performs Bayesian hierarchical regression to estimate malaria parasite clearance rates along with the effect of covariates on them in the presence of “lag” and “tail” phases. In particular, the model performs a linear regression of the log clearance rates on covariates to estimate the effects within a Bayesian hierarchical framework. All posterior inferences are obtained by a “Markov Chain Monte Carlo” based sampling scheme which forms the core of the package.

**Results:**

The “bhrcr” package can be utilized to study malaria parasite clearance data, and specifically, how covariates affect parasite clearance rates. In addition to estimating the clearance rates and the impact of covariates on them, the “bhrcr” package provides tools to calculate the WWARN PCE estimates of the parasite clearance rates as well. The fitted Bayesian model to the clearance profile of each individual, as well as the WWARN PCE estimates, can also be plotted by this package.

**Conclusions:**

This paper explains the Bayesian Clearance Estimator for malaria researchers including describing the freely available software, thus making these methods accessible and practical for modelling covariates’ effects on parasite clearance rates.

## Introduction

In the 1990s, resistance to available anti-malarial drugs such as chloroquine and sulfadoxine–pyrimethamine worsened across areas of the world where malaria is endemic [1]. As a consequence, morbidity and mortality associated with malaria increased, especially among African children, who account for most deaths from malaria [1]. To counteract this, artemisinin-based combination therapy (ACT) was introduced in the mid-1990s. Recent marked increases in the availability and use of ACT, together with the increased use of insecticide-treated bed nets, have substantially reduced global morbidity and mortality from falciparum malaria [2]. However, these gains are threatened by the emergence of artemisinin resistance [3].

Artemisinin resistance can cause the malaria parasites to clear more slowly after treatment and thus slow parasite clearance can indicate resistance. It is worth noting that slow parasite clearance could also be related to host factors such as decreased immunity, inadequate dosing or poor drug absorption. Understanding how covariates relate to parasite clearance rate is important for understanding host and parasite factors’ association with delayed parasite clearance, characterizing resistance and defining spatio-temporal trends in resistance. The parasite clearance rate is defined as the negative of the slope of the log-parasitaemia profile over the time in which the anti-malarial is having its primary effect, where this time period is called the “decay” phase. There are some difficulties that arise in calculating parasite clearance rates. First, some patients’ profiles may contain a “lag” phase, before the decay phase, in which the parasite density remains constant, or even increases, in a period right after drug administration [4, 5]. Second, there might be also a “tail” phase, after the decay phase, where the true parasite count remains close to the detection limit, with no decline over a few measurements, and once the detection limit is reached, observations are left-censored. Lastly, there may exist errors in the measured values of parasite densities (see [6, 7] for more details). The Parasite Clearance Estimator (PCE) was developed by the WorldWide Antimalarial Resistance Network (WWARN) in response to the need from field researchers for a method to quickly and reliably estimate parasite clearance rates, while accounting for the existence of lag phases, tail phases, and censored observations [8].

In some studies, the clearance rates are of interest themselves and for these studies the WWARN PCE is a powerful tool. In other studies, as in [3] and [9], the primary interest in the clearance rates is to understand how they are associated with parasite and host factors; such understanding can provide insights into the mechanism of artemisinin resistance. For these studies, one common approach to estimating the effect of individual level covariates on clearance rates is to use a *two-stage procedure*, where the WWARN PCE is followed by a regression. Even though using the two-stage approach is straightforward, it has some drawbacks. For instance, the WWARN PCE handles profiles with a small number of measurements in a way that can potentially introduce substantial bias in the second-level regression. Additionally, as discussed in [10], the two-stage procedure results in confidence intervals that fail to meet their prescribed coverage guarantees [11], studies a general form of a statistical inference problem involving two components and provides examples in which a two-stage or plug-in procedure performs poorly compared to a full model analysis. These shortcomings of the two-stage approach motivated [10] to develop the Bayesian Clearance Estimator. This procedure uses a Bayesian hierarchical model to estimate both clearance rates and the impact of patient level covariates on them, while accounting for lag phase, tail phase, and censored observations. Simulations in [10] suggest that the Bayesian methodology provides improvements in terms of frequentist properties such as bias and correct coverage of confidence (or credible) intervals. Given the advantages of the Bayesian approach over the two-stage analysis, an **R** [12] package **bhrcr** was built to provide researchers with software that performs the Bayesian hierarchical regression on clearance rates. The **bhrcr** package provides tools to calculate the WWARN PCE estimates of the parasite clearance rates as well.

The rest of the paper is structured as follows. Some fundamental concepts in Bayesian data analysis are first briefly reviewed, as the adopted model falls in the Bayesian statistical inference context. The Bayesian hierarchical regression model introduced and developed by [10] will be then presented. A description of the **bhrcr** package, where the built-in data sets and functions are illustrated by examples, will then follow.

## Bayesian data analysis

In this paper, a Bayesian approach is adopted to build up and implement the model. Before presenting the details of the Bayesian hierarchical regression model, some basic concepts in Bayesian analysis are first briefly reviewed. Many of the following materials in this section are covered in more detail in [13].

**Fig. 1:**
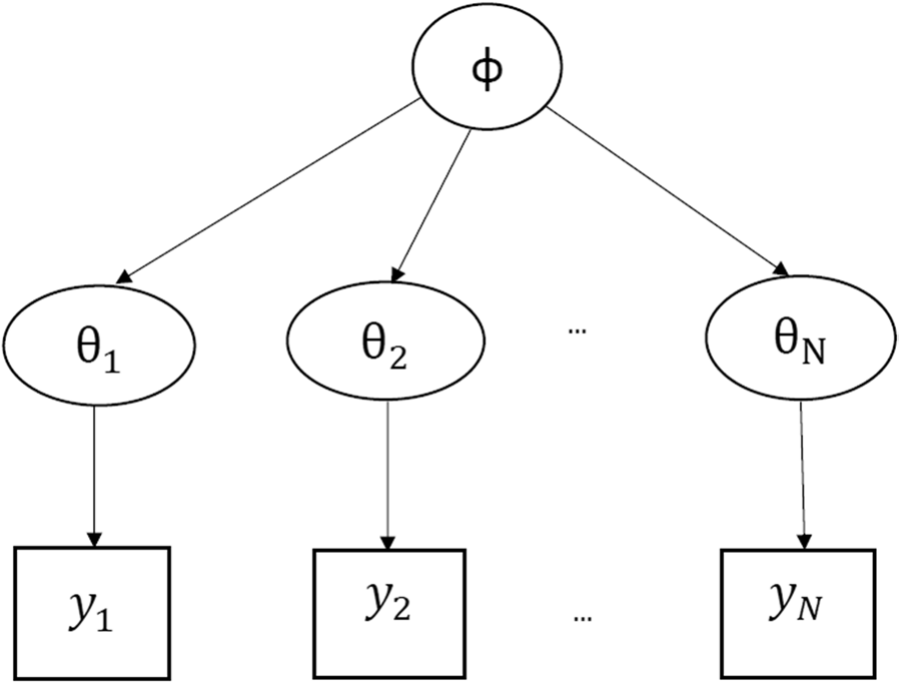
An example hierarchical model where the parameter $\theta _i$ describes the probability distribution of the outcome $y_i$ for subject *i*. In this graphical model, an arrow from *a* to *b* indicates that *b* is generated via *a* through a distribution $p_{a} (b)$, or in other words, *a* describes the distribution of *b*. The main part of this hierarchical model is that $\theta _i$’s are themselves assumed to be draws from a common distribution described by a hyperparameter $\phi$, which within a Bayesian framework, has its own prior distribution

**Fig. 2:**
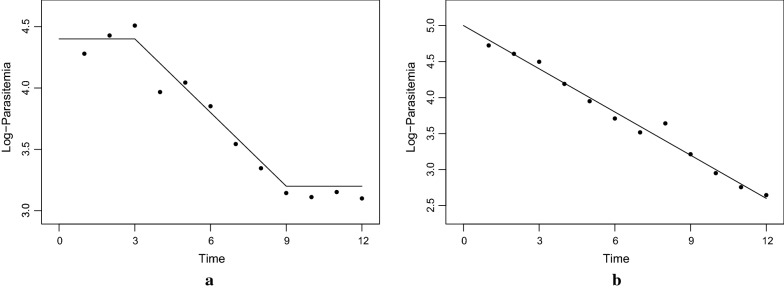
Examples of two profiles along with the true underlying models. **a** has lag, decay, and tail phases with parameters $\delta ^{\ell } = 3, \, \delta ^\tau = 9, \, \beta = 0.2 , \, \alpha = 5$. **b** only has a decay phase and its corresponding parameters are $\delta ^{\ell } = 0, \delta ^\tau = 12, \beta = 0.2, \alpha = 5$

### Bayesian inference

Statistical inference is about drawing conclusions, from numerical data or samples, about quantities that are not observed. As a general notation, let *y* denote the observed data; in this paper’s model, *y* is the malaria parasite densities over time for each patient. Let $\theta$ denote unobservable quantities or population parameters of interest; for example, $\theta$ could include the average half-life of the decay phase and the amount by which different covariates modify this average half-life. While in classical statistics $\theta$ is considered as a fixed unknown, in Bayesian statistical inference, $\theta$ is considered a random variable and inferences about $\theta$ are probability statements conditional on the observed data *y*.

In order to make such a probability statement about $\theta$ given *y*, the first step is to specify a full probability model providing a joint distribution for $\theta$ and *y*. The *joint probability density* function can be written as:

$$\begin{aligned} p(\theta , y) = p(\theta ) \, p(y | \theta ) \end{aligned}$$

where $p(\theta )$ and $p(y | \theta )$ are often referred to as the *prior density* and the *sampling density* or *data likelihood*, respectively. The prior distribution represents the prior belief about the parameters such as the average half life of the decay period. Having specified the prior $p(\theta )$ as well as the likelihood $p(y | \theta )$ which shows how the data is generated, one would then use the property of conditional probability, known as Bayes’ rule, to calculate the *posterior density* of $\theta$:

$$\begin{aligned} p(\theta | y) = \frac{p(\theta , y)}{p(y)} = \frac{p(\theta ) \, p(y | \theta )}{p(y)} \end{aligned}$$

where $p(y) = \int p(\theta )p(y | \theta ) d\theta$ is the marginal density of the data *y*. Since the factor *p*(*y*) does not depend on the unknown $\theta$, it only plays the role of a normalizing constant, and hence, it can be dropped from the above formula without invalidating inferences about $\theta$, in which case one obtains the *unnormalized posterior density*:

$$\begin{aligned} p(\theta | y) \propto p(\theta ) \, p(y | \theta ). \end{aligned}$$

This reveals the relationship that “posterior” is proportional to “prior” $\times$ “data likelihood” in Bayesian statistics. Once $p(\theta | y)$ is calculated (analytically, or numerically in the case where analytical derivations are difficult/impossible), point estimators for $\theta$ can be calculated such as the mean, median or mode of $p(\theta |y)$. 95$\%$ credible intervals for components of $\theta$ are intervals that contain 95$\%$ of the posterior density of those components; typically the central 95$\%$ part of the density. For example, a 95$\%$ credible interval for the average half-life of the decay phase of [12, 18] h would mean that based on the data, there is 95$\%$ probability that the average half-life is between [12, 18] h. Credible intervals are Bayesian analogues of confidence intervals although there are some differences in interpretation.

### Hierarchical models

Many statistical problems involve multiple parameters that can be connected in some way by the structure of the problem. A joint probability model for these parameters should reflect their dependence. It is natural to model such a problem hierarchically, with observable outcomes conditional on certain parameters, which themselves follow a distribution specified by some further parameters, known as *hyperparameters*.

Generally speaking, suppose there are a set of experiments $\{ 1,\ldots ,N \}$, in which experiment *i* is modelled by a likelihood $p(y_i | \theta _i)$ where $y_i$ is the observed data and $\theta _i$ is the unknown parameter. In the case which is of interest in this paper, each patient’s series of measurements of parasite density over time is an experiment (where if there are *N* patients, there are *N* experiments), $y_i$ is the vector of observed parasite densities for patient *i* and $\theta _i$ is the set of parameters which describe the probability distribution of $y_i$ for patient *i*, including the parasite clearance rate, the time of changepoint between the lag and decay phases, and the time of changepoint between the decay and tail phases. Let $\varvec{\theta } = (\theta _1, \ldots , \theta _N)$ represent all parameters in a single vector. The simplest form of a hierarchical model is to let each of the parameters $\theta _i$ be an independent sample from a common distribution $p(\theta | \phi )$ governed by some unknown hyperparameter $\phi$ (see Fig. 1). The hyperparameter $\phi$ describes the distribution of the $\theta _1 ,\ldots ,\theta _N$; for example, it could include the variance of parasite clearance rates over the decay period. By assuming independence, $p(\varvec{\theta } | \phi )$ can be expanded as

$$\begin{aligned} p(\varvec{\theta } | \phi ) = \prod _{i=1}^N p(\theta _i | \phi ) \end{aligned}$$

The key “hierarchical” part is that $\phi$ is not a fixed parameter and thus, in a Bayesian framework, has its own prior distribution $p(\phi )$. Consequently, the joint prior distribution of all unknowns is

$$\begin{aligned} p(\phi , \varvec{\theta }) = p(\phi ) \, p(\varvec{\theta } | \phi ) \end{aligned}$$

and the joint posterior distribution is

$$\begin{aligned} p(\varvec{\theta }, \phi | y) \propto p(\phi , \varvec{\theta }) \, p(y | \phi , \varvec{\theta }) = p(\phi ) \, p(\varvec{\theta } | \phi ) \, p(y | \varvec{\theta }). \end{aligned}$$

Finally, in order to get the marginal posterior of $\theta$ given *y*, $\phi$ must be integrated out:

$$\begin{aligned} p(\varvec{\theta } | y) = \int p(\varvec{\theta }, \phi | y) d\phi . \end{aligned}$$

### Markov chain Monte Carlo (MCMC)

Bayesian inference for hierarchical models is often difficult in practice due to the large number of parameters that commonly appear in a hierarchical model. In general, if the posterior $p(\theta | y)$ cannot be found analytically or if it does not appear to be one of the standard distributions, one may need to draw samples from the posterior distribution through the use of a simulation-based method. Markov chain Monte Carlo (MCMC) is a general simulation method to draw a chain of samples of $\theta$ from the posterior distribution. In the MCMC toolbox, there are some frequently used methods, such as *Gibbs sampling* and *Metropolis-Hastings* algorithms. In the hierarchical regression model introduced in this paper, a combination of samplers known as *Metropolis-Hastings-within-Gibbs* are used to get samples from the posterior distribution. For details of these methods, please see [13].

In MCMC, the longer the chain, the closer the resulting values are to draws from the target distribution that is being estimated. To make the resulting chain more like an independent set of samples, two steps are normally taken. First, a “burn-in” period often needs to be set for the algorithm to discard the first *m* samples. The idea is that a “bad” starting point may over-sample regions that have very low posterior probability before the sampler converges to the target distribution. Hence, the Markov chain needs to be given enough time to reach its equilibrium. Second, MCMC algorithms generate a Markov chain of samples, each of which will be correlated with nearby samples. Thus, if uncorrelated samples are required for inference, one can thin the resulting chain (after the burn-in period) by only taking every *n*-th value, which is called “thinning”.

## Bayesian hierarchical regression on clearance rates

The Bayesian Clearance Estimator developed in [10] is briefly presented in this section. Let $y_{ij}$ represent the *j*th parasitaemia measurement for patient *i* at time $t_{ij}$, where $1 \le i \le N$ and $1 \le j \le n_i$. Suppose $\delta _{i}^{\ell }$ is the time of changepoint between the lag and decay phases for patient *i*, and let $\delta _{i}^\tau$ be patient *i*’s time of changepoint between the decay and tail phases. As the first step in Bayesian analysis, the data likelihood is specified, in which the observed data (in log scale) are assumed to follow a continuous piecewise linear model, where a constant lag phase is followed by a linear decay and a constant tail:

1 $$\begin{aligned} \log (y_{ij}) = \alpha _i - \beta _i \left( \delta _i ^{\ell } \mathbb {1}_{ t_{ij} < \delta _i ^{\ell }} + t_{ij} \mathbb {1}_{ \delta _i ^{\ell } \le t_{ij} \le \delta _i ^\tau } + \delta _i ^\tau \mathbb {1}_{ t_{ij} > \delta _i ^\tau } \right) + \epsilon _{ij} \end{aligned}$$

Note that $\mathbb {1}_A$ is the indicator function of *A* which takes the value one if *A* occurs, and zero otherwise. $\beta _i$ is the clearance rate of the *i*th individual, and the error term $\epsilon _{ij} \overset{iid}{\sim }\mathcal {N}(0 , \sigma _\epsilon ^2)$ ($=$ normal distribution with mean 0 and variance $\sigma _\epsilon ^2$) represents biological variability and measurement error. To further illustrate the model, consider Fig. 2 in which two clearance profiles containing simulated noisy measurements along with the true underlying models are provided. Fig. 2a corresponds to a profile that exhibits lag, decay, and tail phases, with parameters $\delta ^{\ell } = 3$, $\delta ^\tau = 9$, $\beta = 0.2$ (negative of the slope of the decay phase), and $\alpha = 5$. Figure 2b shows a profile with only a decay phase, with parameters $\delta ^{\ell } = 0$, $\delta ^\tau = 12$, $\beta = 0.2$, and $\alpha = 5$.

**Fig. 3:**
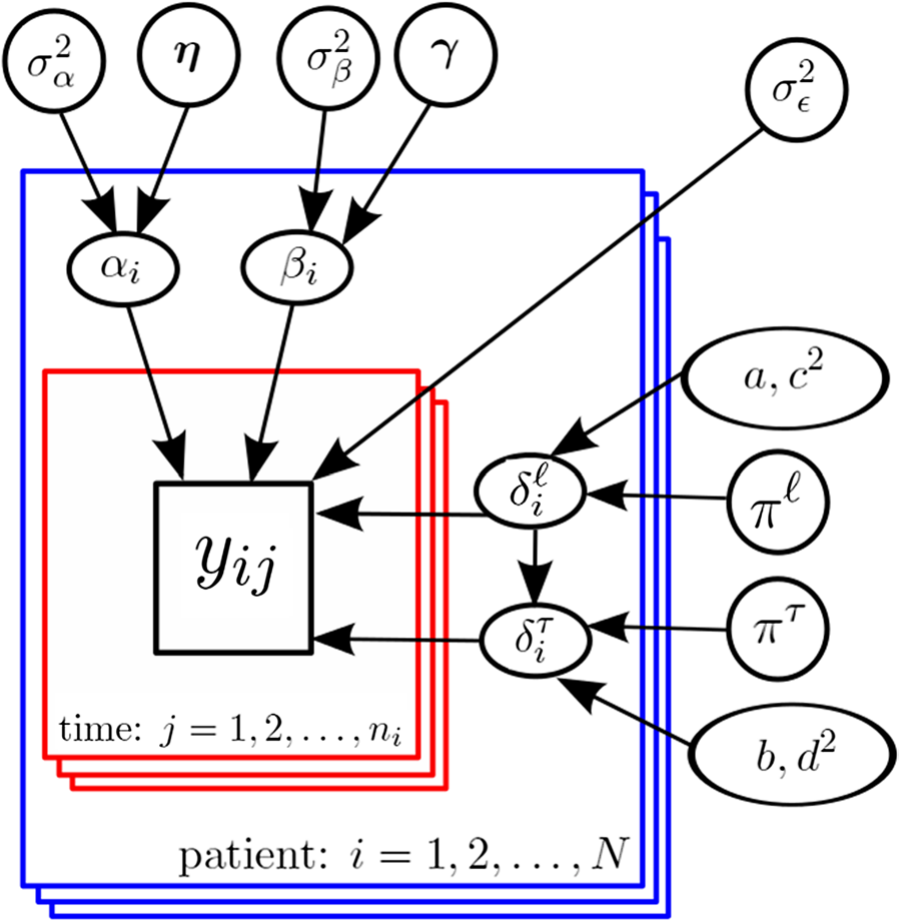
The model hierarchy for the Bayesian Clearance Estimator. Note that each blue box represents a patient and within the blue box of patient *i*, there are $n_i$ red boxes representing her associated parasitemia measurements. In this graphical model, a collection of variables *A* all pointing to a variable *b* simply means the distribution of *b* is described by the variables in *A*. Observe that, following the notation introduced in the previous section, a set of parameters $\theta _i = \{ \alpha _i, \beta _i, \delta _i^{\ell }, \delta _i^\tau \}$ describes the distribution of the measurement vector $\varvec{y}_i = \{ y_{ij} \}_{j=1}^{n_i}$ for patient *i*. Furthermore, each element of $\theta _i$ is assumed to be a sample of a distribution described by the hyperparameters appearing in the figure. For example, $\beta _i$ is assumed to be generated from a distribution which is described by $\gamma$ and $\sigma _\beta ^2$. This distribution and others are all introduced in the text

The second step towards a Bayesian data analysis is specifying the prior distributions for parameters of the model. Within a Bayesian hierarchical structure, the patients’ parameters $\{ \beta _i \}_{i=1}^N$ and $\{ \alpha _i \}_{i=1}^N$, are assumed to be drawn from a common distribution. This hierarchical structure allows one to borrow strength across patients to improve the estimation of patient-specifc parameters. Borrowing strength refers to that, due to regression to the mean, if a patient clears parasites particularly quickly (slowly), it is likely that idiosyncratic factors may have contributed to the patient’s particularly quick (slow) parasite clearance and that the patient’s true parasite clearance rate over many infections would still be quicker (slower) than average but closer to the mean parasite clearance rate. For discussions of borrowing strength, see [13–15] (Chapters 6, 7 and 21, respectively). Figure 3 shows the hierarchical structure embedded in the model where each set of patient-specific parameters are assumed to be draws from a common distribution with some hyperparameters.

Here only the prior on the clearance rates $\{ \beta _i \}_{i=1}^N$ which involves the hyperparameters $\varvec{\gamma }$ and $\sigma _\beta ^2$ is introduced. See the Appendix for the complete set of prior distributions adopted.

Let $\mathbf {X}_i$ be the $1 \times p$ row vector of covariates for patient *i*. The prior on $\beta _i$ is

$$\begin{aligned} \log (\beta _i) \overset{indep.}{\sim }\mathcal {N}(\mathbf {X}_i \varvec{\gamma }, \sigma _\beta ^2) \end{aligned}$$

where $\varvec{\gamma }$ is a $p \times 1$ vector of parameters representing the effect of covariates on $\{ \beta _i \}_{i=1}^N$. Note that $\varvec{\gamma }$ is a parameter of interest in the model, as it represents the impact of covariates on parasite clearance rates. Furthermore, letting $\mathbf {X}_i = 1$ for all *i* corresponds to the case where there are no covariates and estimating the parasite clearance rates based on the Bayesian hierarchical model is of primary interest.

## The **bhrcr** package

The **bhrcr** package takes serial measurements of a response on an individual (e.g., parasite densities after artemisinin administration) over time, and performs Bayesian hierarchical regression on the clearance rates (model shown in Fig. 3). While this tutorial illustrates the method in the context of malaria, the package can be utilized to analyse any clearance data fitting the Bayesian framework presented in the previous section. The *Plasmodium falciparum* clearance data, previously analysed by [9, 10], is included in this package. The main function of the **bhrcr** package is clearanceEstimatorBayes, which will be described thoroughly later on. This function returns the WWARN PCE estimates as well as the estimates from the Bayesian hierarchical model. The calculatePCE function, which provides only the WWARN PCE estimates of the clearance rates, has been incorporated in the package as well. The generic summary, print, and plot functions, as well as the diagnostics function, will also be illustrated by examples in the following subsections.

**Fig. 4:**
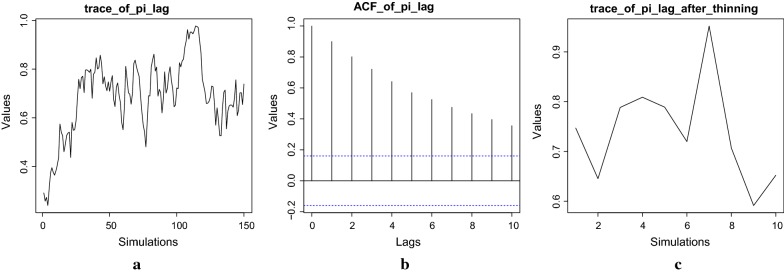
In the fast example burnin = 50, niteration = 100, thin = 10, **a** traceplot of posterior $\pi ^{\ell }$ over the whole 150 simulations; **b** ACF plot; **c** traceplot of thinned posterior samples of $\pi ^{\ell }$

**Fig. 5:**
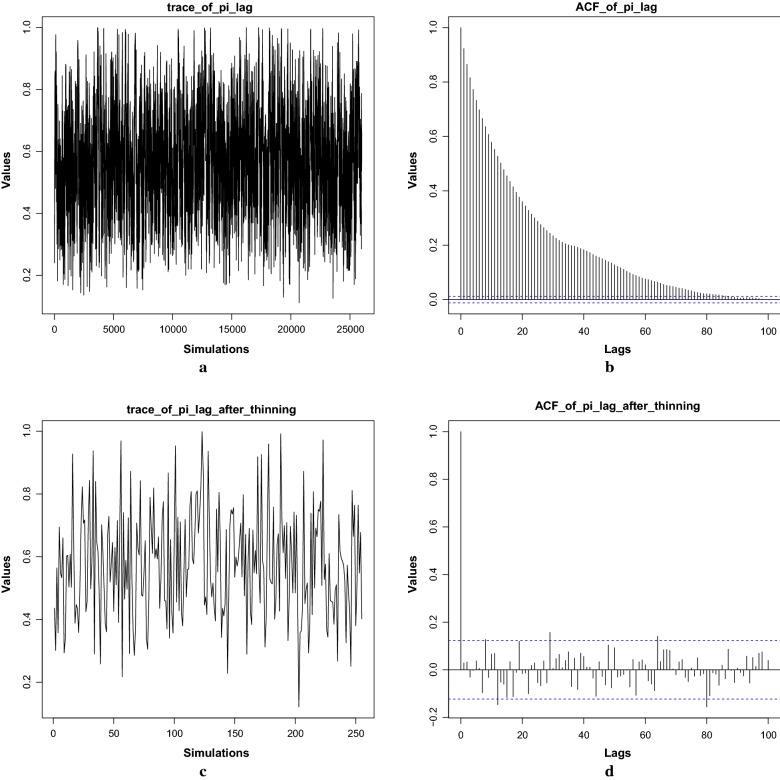
In the slow example burnin = 500, niteration = 25,500, thin = 100, **a** traceplot of posterior $\pi ^{\ell }$ over the whole 26,000 simulations; **b** ACF plot for the whole chain; **c** traceplot of thinned posterior samples of $\pi ^{\ell }$; **d** ACF plot for the thinned chain

To install the package, open a fresh R console and run:

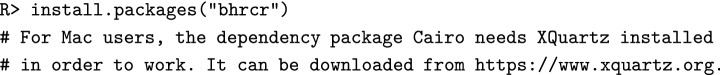



which will automatically download the **bhrcr** package from CRAN and install it on your machine. For a quick demonstration of the package, please run the following functions:

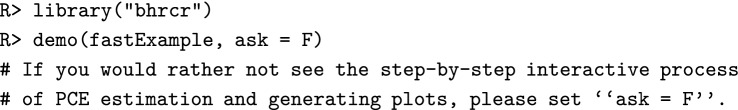



Or one can run the slow example. In the interest of saving the users time, the MCMC in the slow example has already been run for users. The following demo will show you the saved results:

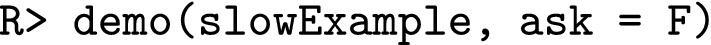



### The Pursat data

The data sets contained in the **bhrcr** package consist of *Plasmodium falciparum* clearance profiles of 110 patients, along with individual level covariates, measured in 2009 and 2010 in the Pursat province of Western Cambodia. Parasite densities were measured every 6 h, and the detection limit was 15 parasites/μl. Additionally, parasites were divided into two genetically different groups, labeled *group 1* and *group 2*. All 110 individuals were observed until no parasites were detected in their blood. The individual level covariates are:

- Sex: A factor variable with two levels F and M
- agegroup: 21+ (21 years of age or older), or 21− (younger than 21 years)
- vvkv: whether or not an individual was from Veal Veng or Kranvanh
- HbE: the number of alleles of Haemoglobin E variant
- athal: the number of alleles of thalassaemia variant
- g6pd: the number of alleles of G6PD deficient variant
- lnPf0: Log initial parasite density
- year2010: TRUE if 2010, FALSE if 2009
- group: 1 if parasite *group 1*, 0 if parasite *group 2*

For more details on the data, see [9, 10]. One can use data(“pursat”) and data(“pursat_covariates”) to access the data sets.

### The clearanceEstimatorBayes function

The clearanceEstimatorBayes function is the principal function in the **bhrcr** package that analyzes the input data set in the Bayesian framework presented in the previous section, and provides the posterior distributions of the parameters, along with point estimates and credible intervals. The arguments of the clearanceEstimatorBayes function as well as their default values and the major components of the function output are explained below:

Usage:

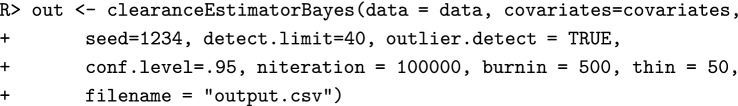



Arguments:

- data: a data frame, with no missing values, containing clearance profiles of patients. This data frame must contain id, time, and count columns, in that order. The first column represents the IDs of patients (not necessarily integers). The second and third columns contain time and recorded parasitaemia (per microlitre) for each of the measurements, respectively. data is allowed to have the predicted WWARN PCE estimates stored in another column named Predicted. If data doesn’t have the Predicted column, clearanceEstimatorBayes will automatically calculate and provide the WWARN PCE rates. In this case it is strongly recommended to set outlier.detect = TRUE. Otherwise, the WWARN PCE outlier detection would not be executed by the program and the provided WWARN PCE rates would be inconsistent with the estimates generated by the online tool.
- covariates: a data frame (with no missing values), ordered according to patients’ order in data, containing individual level covariates. This argument may be NULL, in which case estimation of clearance rates is of primary interest.
- seed: an optional user-specified number used to initialize a pseudorandom number generator, with a default value of 1234. The seed argument helps users to reproduce their results.
- detect.limit: detection limit of the parasite density in blood (parasites per microlitre). The default value is 40.
- outlier.detect: indicator of whether or not to use the WWARN PCE outlier detection method [8]. The default value is TRUE and for the reasons stated before, it is recommended to set outlier.detect = TRUE if data is missing the Predicted column.
- conf.level: required confidence level for reporting estimates’ credible intervals, with a default value of 0.95.
- niteration: total number of simulations after the burn-in period, with a default value of 100,000.
- burnin: length of the burn-in period. The default value is 500.
- thin: step size of the thinning process. The default value is 50.
- filename: the name of the csv file used to store some output elements. This csv file, which is named “output.csv” by default, contains id, clearance.mean, lag.median, and tail.median.

Output: an object of class “bhrcr” containing:

- clearance.post: a matrix of posterior samples for clearance rates $\{ \beta _i \}$.
- clearance.mean: mean values of the clearance rates’ posterior distributions.
- clearance.median: median values of the clearance rates’ posterior distributions.
- gamma.post: a matrix of posterior samples for each element in $\gamma$.
- gamma.mean: mean values of the $\gamma$’s posterior distributions.
- gamma.median: median values of the $\gamma$’s posterior distributions.
- gamma.CI: credible intervals for each element in $\gamma$.
- halflifeslope.post: a matrix of posterior samples for the effect of covariates on log half-lives. The half-life value is calculated as $\log (2)/\text {(clearance rate)}$. Thus, even though the method originally regressed log clearance rates rather than log half-lives on the covariates, one can obtain the slopes for a regression of the log half-lives by using $\log\, (\text {half-life}) = \log \log (2) - \log \,(\text {clearance rate})$.
- halflifeslope.mean: mean values of the posterior distribution for the effect of covariates on log half-lives.
- halflifeslope.median: median values of the posterior distribution for the effect of covariates on log half-lives.
- halflifeslope.CI: credible intervals for the effect of covariates on log half-lives.
- changelag.post: posterior samples of changepoints between lag and decay phases, $\{ \delta _i^{\ell } \}$.
- lag.median: median values of the posterior distributions of $\{ \delta _i^{\ell } \}$.
- changetail.post: posterior samples of changepoints between decay and tail phases, $\{ \delta _i^\tau \}$.
- tail.median: median values of the posterior distributions of $\{ \delta _i^\tau \}$.
- predicted.pce: WWARN PCE estimates of the parasite clearance rates.

This is a partial output list; see clearanceEstimatorBayes man page in the **bhrcr** package for the full list.

### The summary and print functions

The summary function produces comprehensive and compressed output information based on the results from the main function, clearanceEstimatorBayes. To further illustrate this point, the built-in data sets of **bhrcr** package, pursat and pursat_covariates are used to provide a fast example. It may take significant time to run the code, depending on one’s computer’s hardware. Here a small number of iterations is used for tutorial purpose. If the reader wants to obtain stationary results from the simulation, please consider a larger number of iterations. Details will be explained later in the diagnostics function section.

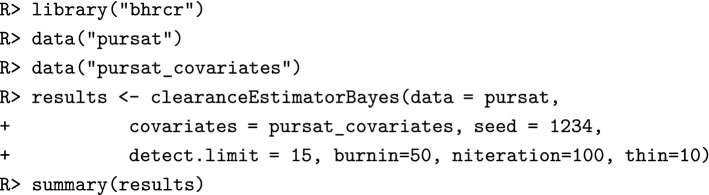



For reproducibility of the results, the seed argument is set to be 1234. The output given by summary includes a table containing the posterior mean and median of the regression coefficients which represent the impact of covariates on log parasite clearance rates and also on the corresponding log half-life values, along with the 95% credible intervals. If the input data set does not contain WWARN PCE estimates, the clearanceEstimatorBayes function will automatically generate a folder called “PceEstimates” under your current working directory to store calculated WWARN PCE estimates for each individual.

In what follows, the results are displayed in terms of log half-lives which may be more intuitive to the malaria research community. The half-life is the time it takes for the parasite density to reduce by 50%; the longer the half-life, the slower the parasite clearance.

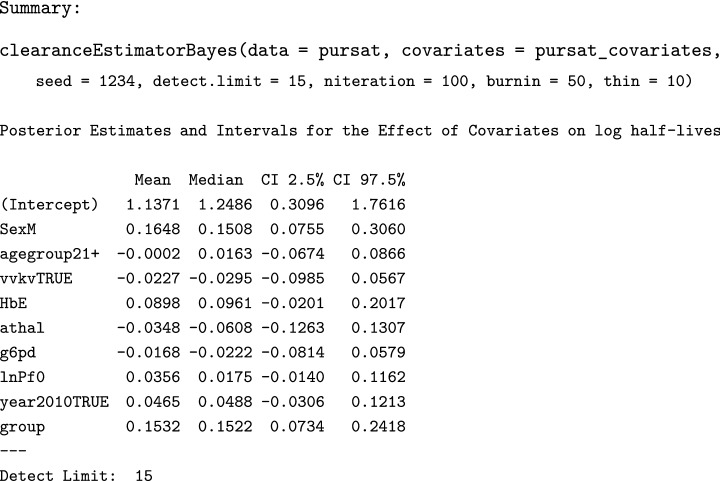



Based on the output of the summary function, one can perform an analysis of the covariates of interest. As discussed in Section 4 of [10], one point of interest was whether or not there is evidence of resistance to artemisinins developing over time. Thus the indicator variable year2010TRUE for the year of data collection was included. According to the results above, the parasite clearance half-life increased over time (positive mean and median) however this effect is not significant since its 95% credible interval contains zero.

One may also want to know whether certain aspects of host genetics impact the resulting half-lives. There is a hypothesis given in [9] that red blood cell polymorphisms—including Haemoglobin E (HbE), thalassaemia (athal), and G6PD deficiency (g6pd)—may act to strengthen the pro-oxidant activity of parasite defenses against artemisinins, hence resulting in lower clearance rates. From the results in the example, none of these factors has a significant positive impact on log half-lives since the 95% credible intervals all contain 0. For a detailed posterior analysis based on longer Markov chains, please see Section 4 in [10].

Finally, one may be also interested in how acquired immunity to the effects of *Plasmodium falciparum* may impact half-lives. In the analysis, three covariates were included that are surrogates for increased likelihood of exposure to malaria: male gender (SexM), age 21 or greater (agegroup21+) and living in the Kravanh or Veal Veng districts (vvkvTRUE) which are close to forested regions (see [9]). Notice the slope of 0.1648 on the indicator variable SexM for males which means that parasite clearance half-life is estimated to be longer in male patients than in female patients, other factors in the model being held equal, by a factor of $e^{0.1648}\approx 1.179$. But one should be careful about the interpretation here because this is an observational study and there may be unmeasured confounders. The causal interpretation of each covariate is not straightforward and more or less speculative. The reader can refer to [16] for some details.

The print function is essentially the same. It only displays the posterior mean of the effect of covariates on both log clearance rates and log half-lives. Therefore, for a quick and straightforward summary of the estimated impact of covariates, the print function is recommended.

### The diagnostics function

The diagnostics function provides diagnostic analysis such as trace plots, ACF (auto-correlation function) and PACF (partial auto-correlation function) plots for some important parameters in the MCMC process of the Gibbs sampling. These diagnostic plots help to assess whether it is plausible that the MCMC process has reached stationarity and has been thinned sufficiently (see [17, 18]).

Here the previous results are used as an example. All diagnostic plots will be saved under “./mcmcDiagnostics”.

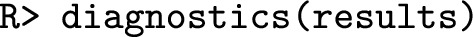



In the fast tutorial example, the burn-in period and the total length of simulation (also referred to as the length of Markov chain) are short, which may not provide enough time for convergence. For serious malaria research, here are some recommendations:

1. Detect outliers by using the methodology suggested in [8]. Flegg’s outlier detection method is recommended. However, users can choose to toggle it off by setting outlier.detect = FALSE when they are running the main function clearanceEstimatorBayes. If the outliers are determined to be likely due to transcription errors, then the outlying data points should be deleted;
2. Run the MCMC algorithm (already embedded in clearanceEstimatorBayes) with various lengths and observe the trace plots, ACF plots (explained later), which helps determine the suitable burn-in period. Make sure the final sample is collected after the Markov chain reaches stationarity, i.e. the distribution of the values after the burn-in ends should be similar to the values at the middle and end of the chain. For the current version of bhrcr, parallelization is not supported so that users have to run one chain at a time;
3. Run the formal MCMC with a long run instead of just several short runs. Only a long run can give the Markov chain enough time to mix well and thus to get its equilibrium since one is not able to foresee how slow the mixing rate might be for real problems especially for those in high-dimensional space;
4. Optional: set a suitable step size in “thinning” to make sure the final sample is close to independent if independence or low correlation is highly desired (the ACF plot can be used to detect autocorrelation). But “thinning” will inevitably sacrifice some estimation efficiency.

The above steps will be further explained below to show how to analyse the posterior samples from MCMC by using the fast and slow examples in the **bhrcr** package.

Here only one set of trace results is displayed. Figure 4 shows diagnostic plots corresponding to the parameter $\pi ^{\ell }$ in the fast example. These plots are cause for concern. The traceplot over the whole simulation (including the burn-in period) is shown in Fig. 4a. The massive oscillations in the traceplot make it nearly impossible to ascertain whether or not stationarity has been attained over the course of the chain, giving no satisfactory choice of burnin. The ACF plot in Fig. 4b shows that significant autocorrelation exists in the candidate posterior sample. Note the blue dotted lines give the confidence interval beyond which the autocorrelations are (statistically) significantly different from zero. In Fig. 4b, the autocorrelations are slowly decaying instead of dropping to zero (within the blue dotted lines) after small lags. The traceplot after a burnin and thinning, shown in Fig. 4c, is utterly uninformative for assessing convergence and stationarity of the resulting chain. Because, after burn-in and thinning, there are only $(150 - 50)/10 = 10$ samples which is too small for accurate inference. For a more informative plot after thinning, please check Fig. 5c in the slow example. In conclusion, for the fast sample, the number of resulting iterations is clearly too few to result in a satisfactory posterior sample. In order to determine the suitable number of simulations, a sequential strategy could be used in which one first tries a number of posterior samples and checks whether convergence has been achieved, and if it has not, then one takes more posterior samples. As a first try in the sequential strategy, at least 200 and preferably 1000 samples are recommended. Since the fast sample involves considerably less samples, the posterior results produced by the fast example may not be very reliable; the fast sample is used only for tutorial purposes.

For the results of the Bayesian clearance estimator to truly reflect the posterior uncertainty in the estimators, one needs to be confident that stationarity has been achieved. Results that satisfy the requisite diagnostics are found in a longer sample (slowExample), which has been saved into a dataset called posterior.rda and incorporated into the **bhrcr** package. To see the results, run the slow example in the demo:

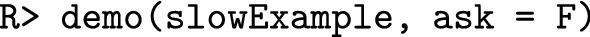



Figure 5 shows one set of plots related to the parameter $\pi ^{\ell }$. According to Fig. 5a, there is no long-term trend in the trace plot and the average value seems to be flat, which suggests the Markov chain has reached stationarity. But Fig. 5b indicates that it has very high-order autocorrelation since the plot shows significant exponential decay autocorrelation values persisting over a long period of lags which is a typical behaviour of the AR (Auto-Regressive) model. After burn-in and thinning (see Fig. 5c, d), a stationary thinned chain with uncorrelated nearby samples appears to have been obtained since its ACF exhibits a sharp cutoff after lag 0.

Gelman and Rubin [19] suggest running MCMC simulations multiple times with different configurations and observing whether the within chain variation is similar to the between chain variation as a way of assessing MCMC convergence. Practitioners should take this advice. In the previous work [10], a Metropolis-Hasting-within-Gibbs sampling algorithm on this data set from six different starting locations was run, for 50,500 iterations (with 500 iterations as burn-in) per starting location. As such, each chain was thinned by only keeping one out of every 100 iterations. The six chains in total provided 3000 roughly independent posterior samples.

### The plot function and posterior analysis

The plot function visualizes the results returned by the clearanceEstimatorBayes function. All plots will be saved under “./plots”. The previous example is used as follows.

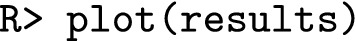



**Fig. 6:**
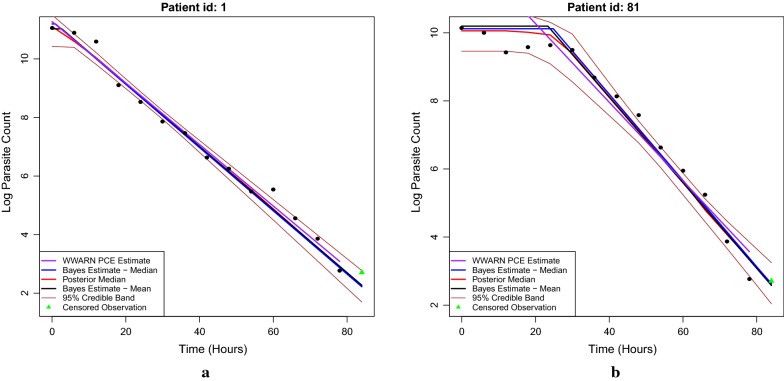
**a** The posterior log-parasitaemia profile of patient 1. The profile of patient 1 is identified as only having a decay phase. The brown lines characterize the point-wise 95% credible intervals of the posterior samples. The solid black and blue lines represent, respectively, the posterior mean and median clearance rates from the Bayesian procedure. The solid red line is the posterior median curve and the purple line is the fit given by the WWARN PCE method. The triangles are censored observations due to the detection limit. **b** The posterior log-parasitaemia profile of patient 81. The profile of patient 81 is identified as having a significant lag phase before the decay occurs. The solid black and blue lines (in the decay phase) represent the posterior mean and median clearance rates from the Bayesian procedure, respectively. The solid red line is the posterior median curve and the purple line is the fit given by the WWARN PCE method. The green triangles are censored observations due to the detection limit

The output provides a group of figures showing each patient’s posterior log-parasitaemia profiles fitted by the Bayesian method. Figure 6a shows an individual whose profile seems to exhibit only a decay phase, whereas Fig. 6b shows an individual who is identified as having a lag phase before the decay occurs.

By using the following commands, one can calculate the posterior mean, median, and 95% credible interval of each individual’s clearance rate. Several specific individuals can be picked by using a vector of IDs. In the following example, one can check patients with ID “1”, “3”, “14”, “35”. Here the ID numbers are stored as string/characters instead of numeric integers. This allows for general use of extracting specific patients in terms of given IDs such as names or bar code sequence etc.

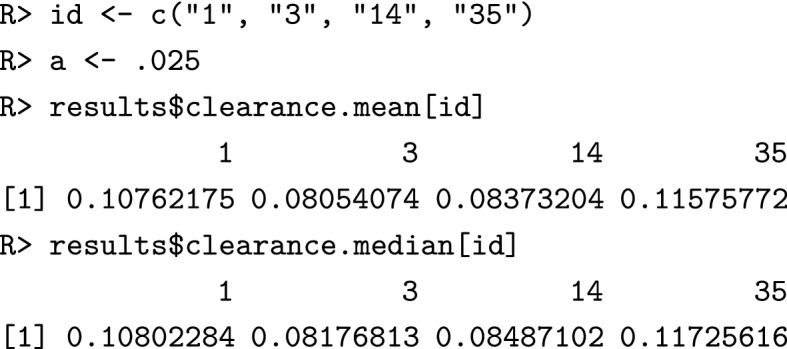



If one wants to check several patients’ credible intervals simultaneously,

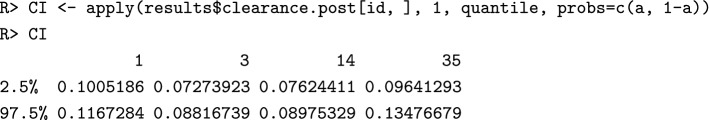



If one wants to check only one patient’s credible interval, for instance patient id = 1,

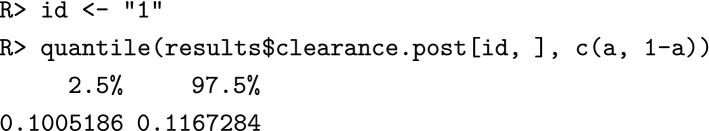



For the patient with id 1 (see Fig. 6a), the posterior mean clearance rate was 0.1076, the median was 0.1080 with a 95% credible interval of [0.1005, 0.1167]. For this patient, one can check the posterior distribution of the time of the changepoint between the lag and decay phases:

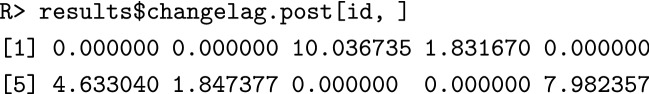



The output is a vector of posterior samples of changepoint time. There are 10 posterior samples in total after thinning for the fast example. Only 20% of the posterior samples identified this individual as having a lag phase of more than 6 h, and only 30% identified a lag phase of more than 3 h. The analysis here is based on the previous fast example which has a small number of total iterations. So the posterior results are only used here for tutorial purposes.

For the individual in Fig. 6b (with id 81), the posterior mean clearance rate was 0.1284, the median was 0.1270 with a 95% credible interval of [0.1196, 0.1423]. There are 100% posterior of samples identifying this individual as having a lag phase of greater than 6 h, whereas no samples identified a tail phase, as shown by

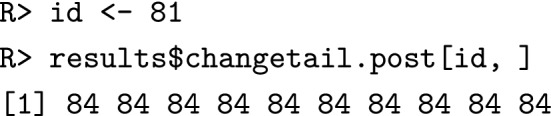



which implies that a tail phase was not observed in any posterior sample. The posterior median of the time of changepoint between lag and decay phases for this individual is 24.86 (h), which can be obtained by

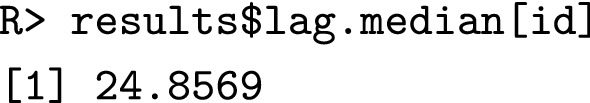



The 95% credible interval for the time of changepoint between lag and decay phases is [8.337287, 28.843629], which can be obtained by:

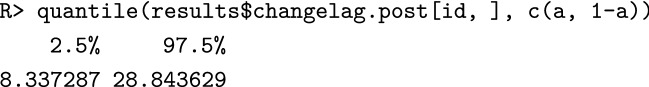



Last but not least, there are four different posterior curves produced by the plot function: the mean (coefficient) curve, the median (coefficient) curve, the posterior median curve and the point-wise 95% credible intervals of the posterior samples. In Fig. 6a, b,

- The “mean curve” is obtained by plugging the posterior mean values of all coefficients into the change-point model (Eq. 1) which is displayed as the black piece-wise linear curves in Fig. 6a as well as in Fig. 6b;
- The blue “median curve” is produced similarly by plugging the posterior median values of all coefficients into the change-point model (Eq. 1);
- The “posterior median curve” is obtained by taking the timepoint-wise median of all the posterior sample curves (not shown in Fig. 6). This curve is shown in red in Fig. 6a, b. Note that a “posterior mean curve” is not included since due to the linearity of expectation, the “posterior mean curve” would be the same as the black “mean curve”.
- The point-wise 95% credible intervals of the posterior samples are calculated at each time point by using all the posterior samples, which are shown as the brown lines (upper bound and lower bound).

All these curves are available in the plot function to give users more flexibility to choose what they prefer.

## Discussion

The **bhrcr** package is quite general, in that, given any data set which is expected to follow linear decay possibly with lag and/or tail phases, it can produce the Bayesian hierarchical estimates of the clearance rates together with regression analysis on interesting covariates and data visualization. The package makes the Bayesian hierarchical clearance rate regression method developed in [10] much more accessible to the malaria research community. In this paper, a fast example with a small number of burn-in periods and iterations in the MCMC process was illustrated, which may lead to non-stationarity since according to Section 4 in [10], the convergence rate is quite slow. This paper serves as a tutorial for the **bhrcr** package and introduces the basic functions it provides. It is hoped that the **bhrcr** package will be useful to the malaria research community and beyond for investigating parasite clearance rates.

## Appendix

Here the adopted prior distributions and the implementation of the model are briefly outlined:

- Let $\mathbf {X}_i$ be the $1 \times p$ row vector of covariates for patient *i*. The priors on $\alpha _i$ and $\beta _i$ are
  $$\begin{aligned} \log (\beta _i) \overset{indep.}{\sim }\mathcal {N}(\mathbf {X}_i \varvec{\gamma }, \sigma _\beta ^2), \quad \log (\alpha _i) \overset{indep.}{\sim }\mathcal {N}(\mathbf {X}_i \varvec{\eta }, \sigma _\alpha ^2) \end{aligned}$$
  where $\varvec{\gamma }$ and $\varvec{\eta }$ are $p \times 1$ vectors of parameters representing the effect of covariates on $\{ \beta _i \}_{i=1}^N$ and $\{ \alpha _i \}_{i=1}^N$, respectively.
- Let $\pi ^{\ell }$ and $\pi ^\tau$ be a priori probabilities of there being a lag and a tail phase respectively. The prior distributions on $\delta _i^{\ell }$ and $\delta _i^\tau$ are
  $$\begin{aligned} \delta _i ^{\ell } | \pi ^{\ell }, \pi ^\tau , a, c^2 \sim \pi ^{\ell } \mathcal {L}\mathcal {N}(a,c^2) \mathbb {1}_{\delta _i ^{\ell } < t_{i n_{i}} } + (1 - \pi ^{\ell }) \mathbb {1}_{\delta _i ^{\ell } = 0 } \end{aligned}$$
  $$\begin{aligned} (t_{i n_{i}} - \delta _i ^\tau ) | \delta _i ^{\ell }, \pi ^{\ell }, \pi ^\tau , b, d^2 \sim \pi ^\tau \mathcal {L}\mathcal {N}(b,d^2) \mathbb {1}_{\delta _i ^\tau > \delta _i ^{\ell } } + (1 - \pi ^\tau ) \mathbb {1}_{\delta _i ^\tau = t_{i n_{i}} } \end{aligned}$$
  where $\mathcal {L}\mathcal {N}(\mu , \sigma ^2)$ (log-normal) is a random variable whose logarithm is normally distributed with parameters $\mu$ and $\sigma ^2$.
- See [10] for priors on the model’s hyperparameters $\sigma _\epsilon ^2$, $\{ \varvec{\gamma }, \sigma _\beta ^2 \}$, $\{ \varvec{\eta }, \sigma _\alpha ^2 \}$, $\pi ^{\ell }$, $\pi ^\tau$, *a*, *b*, $c^2$, and $d^2$.
- A Metropolis-Hastings-within-Gibbs sampling algorithm is used to obtain samples that are approximately from the posterior distribution.
